# Disparities in Use of Video Telemedicine Among Patients With Limited English Proficiency During the COVID-19 Pandemic

**DOI:** 10.1001/jamanetworkopen.2021.33129

**Published:** 2021-11-04

**Authors:** Loretta Hsueh, Jie Huang, Andrea K. Millman, Anjali Gopalan, Rahul K. Parikh, Silvia Teran, Mary E. Reed

**Affiliations:** 1 Division of Research, Kaiser Permanente Northern California, Oakland; 2 The Permanente Medical Group, Oakland, California

## Abstract

This cross-sectional study assesses whether having limited English proficiency was associated with lower video use compared with telephone use, especially among patients without prior video visit experience, during the COVID-19 pandemic.

## Introduction

Telemedicine expands health care access for patients facing barriers to in-person care,^1^ but may also inadvertently widen existing care disparities^2,3^ for the 25 million people living in the US with limited English proficiency (LEP)^4^ because of overlapping low digital literacy and health literacy.^5^ Data on differential video vs telephone visit use by patients with LEP are needed to inform telemedicine equity strategies. In patients self-scheduling a primary care visit during the COVID-19 pandemic, we hypothesized that LEP would be associated with lower video use compared with telephone, especially among patients without prior video visit experience.

## Methods

The retrospective cross-sectional study received institutional review board approval at Kaiser Permanente Northern California (KPNC) and waived informed consent because this was a data-only study with no participant contact. We followed the Strengthening the Reporting of Observational Studies in Epidemiology (STROBE) reporting guideline.

This study included all patient portal self-scheduled primary care telemedicine visits within KPNC from March 16 to October 31, 2020. In-person visits were only available by clinician recommendation after an initial telemedicine visit. Visits were accessible via any internet-enabled device. We extracted patient sociodemographics, technology access factors, and whether the patient visited their own primary care physician from automated data sources. Multivariate analyses examined the association between scheduling a video visit (vs telephone) and LEP, which was defined as needing an interpreter. An LEP × prior video visit interaction term was added to the multivariate regression to examine whether barriers to the initial video visit can potentially explain video visit use differences by LEP (ie, no video visit use differences by LEP among those with prior video visit use experience would suggest patients with LEP were not dissuaded by their initial video visit experiences).

We report adjusted video visit use frequencies generated from model coefficients for comparisons using Stata, version 14.2 (StataCorp LLC). Two-sided χ^2^ tests were used to calculate *P* values for patient sociodemographics, technology access, and provider factors for interpreter need (Table 1). Two-sided logistics regressions were used to calculate *P* values for odds ratios from multivariable model of the association between the patient’s need for a language interpreter and video vs telephone telemedicine visit. Statistical significance was set at *P* < .05. Data were analyzed between February and April 2021.

**Table 1.** Patient Sociodemographics, Technology Access, and PCP Factors by Interpreter Need

Characteristics	No. (%)		
	Total sample (N = 955 352)	Interpreter need^**a**^	
		No (n = 932 876)	Yes (n = 22 476)
Race and/or ethnicity			
Asian	216 788 (22.7)	205 731 (94.9)	9587 (4.9)
Black	70 281 (7.4)	70 208 (99.9)	73 (0.1)
Hispanic/Latino	196 483 (20.6)	186 896 (95.1)	9587 (4.9)
White	454 741 (47.6)	453 189 (99.7)	1552 (0.3)
Another race and/or ethnicity	14 796 (1.6)	16 661 (99.1)	135 (0.9)
Unknown	2265 (0.2)	2191 (96.8)	72 (3.2)
Age			
<18	106 353 (11)	103 671 (11.1)	1156 (5.1)
18-64	720 338 (74.5)	695 623 (74.6)	15 506 (69)
≥65	140 173 (14.5)	133 582 (14.3)	5814 (25.9)
Men	409 632 (42.4)	395 027 (42.4)	8771 (39)
Women	545 720 (57.1%)	537 849 (57.7)	13 705 (61.0)
Low neighborhood SES^b^	195 612 (20.2)	185 122 (19.8)	8217 (36.6)
Technology access factors and visit with own PCP			
Low neighborhood internet access^c^	387 444 (40.1)	371 647 (39.8)	11 490 (51.1)
Prior video visit experience^d^	280 879 (29.1)	273 316 (29.3)	5439 (24.2)
Prior mobile portal access^d^	381 746 (39.5)	366 963 (39.3)	8950 (39.8)
Visit with own PCP	734 474 (76)	708 358 (75.9)	17 738 (78.9)

Abbreviations: PCP, primary care provider; SES, socioeconomic status.

^a^ Patients with interpreter need differed from patients without interpreter need on all study covariates at *P* < .001.

^b^ Neighborhood SES was calculated using 2010 US census measures at the census block group level; low SES was determined if 20% or more of residents have household incomes below the federal poverty level or 25% or more of residents 25 years of age or older have less than a high school education.

^c^ Low neighborhood internet access level was defined as having less than 80% of households with a residential fixed high-speed connection at least 10 megabits per second downstream and at least 1 megabits per second upstream in the given census tract.

^d^ Prior video visit experience and prior mobile portal access is past 12 months. Prior mobile portal access was considered a proxy for technology access and comfort with technology but was not required to conduct a video visit.

## Results

Among 955 352 primary care telemedicine visits (video: 379 002 [39.6%]; telephone: 576 350 [60.3%]) scheduled by 642 370 patients. There were 22 476 (2.4%) with EHR-documented interpreter need, 454 741 (47.6%) White patients, 216 788 (22.7%) Asian patients, and 196 483 (20.6%) Hispanic patients; 720 338 (74.5%) patients aged 18 to 64 years, 409 632 (42.4%) men, and 195 612 (20.2%) patients from low SES neighborhoods (Table 1). Patients with LEP used video visits less frequently (7765 [34.5%]) than patients without LEP (371 237 [39.8%]). After multivariate adjustment, LEP vs no LEP was associated with lower video visit use (OR, 0.77; 95% CI, 0.74-0.80; adjusted video visit frequency of 34.7% for LEP vs 39.8% for no LEP) (Table 2).

**Table 2.** Odds Ratios From Multivariable Model of the Association Between Patient’s Need for Language Interpreter and Video (vs Telephone) Telemedicine Visit

Characteristics^a^	OR (95% CI)	*P* value
Interpreter need	0.77 (0.74-0.80)	<.001
Patient sociodemographics		
Race/ethnicity		
White	1 [Reference]	NA
Black	0.87 (0.85-0.89)	<.001
Hispanic/Latino	0.82 (0.81-0.83)	<.001
Asian	1.20 (1.19-1.22)	<.001
Another race/ethnicity	0.92 (0.88-0.96)	<.001
Unknown	0.72 (0.65-0.79)	<.001
Age		
18-64	1 [Reference]	NA
<18	1.79 (1.76-1.82)	<.001
≥65	1.29 (1.27-1.31)	<.001
Men	1.13 (1.11-1.14)	<.001
Low neighborhood SES	0.83 (0.82-0.84)	<.001
Technology access and PCP factors		
Low neighborhood internet	0.91 (0.90-0.92)	<.001
Mobile portal use	1.13 (1.12-1.15)	<.001
Prior video visit experience	1.76 (1.74-1.78)	<.001
Visit with own PCP	1.15 (1.14-1.17)	<.001

Abbreviations: NA, not applicable; OR, odds ratio; PCP, primary care physician; SES, socioeconomic status.

^a^ All models adjusted for time, medical service area (to account for clinic-level variation), *International Statistical Classification of Diseases and Related Health Problems*, *Tenth Revision* grouping to account for comorbidities and account for clustering within patients. The total number of participants was 955 352.

The association between LEP and visit type differed by prior video visit experience (*P* < .001 for interaction). Simple effects tests and adjusted video visit frequencies showed that among patients without prior video visit experience, adults with LEP vs without LEP were less likely to use video visits (28.9% vs 35.9%; *P* < .001). However, among patients with prior video visit experience, adults with LEP did not differ from adults without LEP in their likelihood of choosing video visits (47.2% vs 49.1%; *P* = .09).

## Discussion

In this large, diverse sample of patients seeking primary care during the COVID-19 pandemic, one-third of patients with LEP scheduled a visit by video instead of telephone. Patients with LEP chose video less often than patients without LEP, even after adjusting for technology factors. However, among patients with video visit experience, no significant difference in video visit use by LEP was found, suggesting that once patients with LEP have video visit use experience, they are not different from patients without LEP in likelihood to reuse video visits. Although our analyses cannot determine causality, it is reasonable to hypothesize that helping adults with LEP overcome initial barriers to using video visits will result in more frequent future video visit use.

Our reliance on EHR-documented interpreter need as a proxy for LEP is a key study limitation. Nonetheless, with the rapid expansion and likely persistence of video telemedicine for delivering primary care, additional research is needed to identify barriers to initial video telemedicine use among patients with LEP.
